# The association between being currently in school and HIV prevalence among young women in nine eastern and southern African countries

**DOI:** 10.1371/journal.pone.0198898

**Published:** 2018-06-20

**Authors:** Paul Mee, Elizabeth Fearon, Syreen Hassan, Bernadette Hensen, Xeno Acharya, Brian D. Rice, James R. Hargreaves

**Affiliations:** 1 MeSH Consortium, Department of Public Health Environments and Society, Faculty of Public Health and Policy, London School of Hygiene and Tropical Medicine, London, United Kingdom; 2 Centre for Evaluation, London School of Hygiene and Tropical Medicine, London, United Kingdom; 3 Department of Public Health Environments and Society, Faculty of Public Health and Policy, London School of Hygiene and Tropical Medicine, London, United Kingdom; 4 Clinical Research Department, Faculty of Infectious and Tropical Diseases, London School of Hygiene and Tropical Medicine, London, United Kingdom; UNAIDS, UNITED STATES

## Abstract

**Introduction:**

Interventions to keep adolescent girls and young women in school, or support their return to school, are hypothesised to also reduce HIV risk. Such interventions are included in the DREAMS combination package of evidence-based interventions. Although there is evidence of reduced risky sexual behaviours, the impact on HIV incidence is unclear. We used nationally representative surveys to investigate the association between being in school and HIV prevalence.

**Methods:**

We analysed Demographic and Health Survey data from nine DREAMS countries in sub-Saharan Africa restricted to young women aged 15–19 (n = 20,429 in total). We used logistic regression to assess cross-sectional associations between being in school and HIV status and present odds ratios adjusted for age, socio-economic status, residence, marital status, educational attainment and birth history (aOR). We investigated whether associations seen differed across countries and by age.

**Results:**

HIV prevalence (1.0%–9.8%), being currently in school (50.0%-72.6%) and the strength of association between the two, varied between countries. We found strong evidence that being currently in school was associated with a reduced odds of being HIV positive in Lesotho (aOR: 0.37; 95%CI: 0.17–0.79), Swaziland (aOR: 0.32; 95%CI: 0.17–0.59), and Uganda (aOR: 0.48: 95%CI: 0.29–0.80) and no statistically significant evidence for this in Kenya, Malawi, Mozambique, Tanzania, Zambia or Zimbabwe.

**Conclusions:**

Although the relationship is not uniform across countries or over time, these data are supportive of the hypothesis that young women in school are at lower risk of being HIV positive than those who leave school in some sub-Saharan African settings. There is a possibility of reverse causality, with pre-existing HIV infection leading to school drop-out. Further investigation of the contextual factors behind this variation will be important in interpreting the results of HIV prevention interventions promoting retention in school.

## Introduction

Among the general population in sub-Saharan Africa, young women aged 15 to 24 are at a high risk of HIV infection, with 25% of new infections occurring among this group in 2015 [1]. Young women’s increased risk of HIV acquisition is due to a complex interplay of biological and behavioural factors. Examples include the increased risk of infection associated with each sex act for females compared to males [2], the occurrence of age-disparate sexual relationships [3, 4], and more distal structural factors related to socio-economic and cultural inequity between genders [5]. To address these factors, the DREAMS (“Determined, Resilient, Empowered, AIDS-free, Mentored and Safe”) initiative is implementing a combination package of evidence-based interventions in ten sub-Saharan African countries to reduce HIV incidence by 40% among adolescents girls and young women [6]. The DREAMS initiative combines the delivery of technologies known to prevent HIV, including condoms, with interventions to address structural factors that influence HIV risk [6].

To date there has been mixed observational evidence on the association between educational attainment and HIV status across sub-Saharan Africa [7–9]. Findings from studies assessing interventions to increase school attendance have also been mixed, though in many cases promising. Two quasi-experimental studies from Botswana [10] and Malawi and Uganda [11] investigating the impact of changes in national educational policy presented evidence for a decrease in the probability of testing positive for HIV with each additional year of education gained. Further studies in sub-Saharan Africa have shown increased levels of school attendance to be associated with lower risk sexual behaviour [12] [13], providing evidence for a pathway that would explain the causal mechanisms for a protective effect. A trial in Malawi showed a decrease in HIV prevalence among young women receiving a cash incentive conditional on school attendance [14]. A South African randomised trial, which investigated whether providing cash incentives for school attendance reduced HIV incidence, found that young women who had lower school attendance or those who dropped out of school had a higher risk of being HIV positive [15] [16].

There are different mechanisms by which education could be protective against the acquisition of HIV. More time in school could lead to higher exposure to sexual and reproductive health education[17]. Accumulating higher levels of education and associated qualifications could improve the young women’s socio-economic position, leaving them less dependent on sexual partners and more empowered to negotiate safer sexual practices such as condom use. Additionally, gaining higher levels of education may lead to the development of stronger socio-cognitive abilities and therefore the ability to better assimilate risk information [6]. It is also possible that education is protective because young women are spending a large proportion of their time in school, in a social environment of fellow students, rather than outside of school in an environment in which they might be more likely to meet partners from whom they are more likely to acquire HIV [18].

Important components of the DREAMS package are interventions such as cash transfers or educational subsidies to support retention and promote return to school [6]. To date, much of the available evidence explores the relationship between educational attainment and HIV. In this study, we use nationally representative surveys to explore the association between currently being in school and prevalent HIV, controlling for the effect of potential confounders. We explore how this association varies across the DREAMS countries and whether it changes with age.

## Methods

### Study setting and populations

Data on socio-demographic factors and HIV prevalence among females aged 15–19 years was extracted from the most recently available data from nationally representative population-based Demographic and Health surveys (DHS) (http://dhsprogram.com/). These were conducted in nine of the ten countries included in the DREAMS initiative: Kenya (2008–09), Lesotho (2014), Malawi (2015–16), Mozambique (2009), Uganda (2011), Tanzania (2011–12), Swaziland (2006–07), Zambia (2013–14) and Zimbabwe (2015) [19–27]. The remaining DREAMS country, South Africa, does not conduct DHS surveys. The study population comprised those who had an HIV test result recorded and current education status available. The study population was 20,429. (Table 1). Study procedures and questionnaires for DHS surveys are approved by the ICF Institutional Review Board (IRB) and individual IRBs in the host countries. This study has been reviewed and approved by the London School of Hygiene and Tropical Medicine Observational Research Ethics committee. The protocols for conducting DHS surveys include rigorous procedures to ensure data remains fully anonymised, both at an individual level and spatially. Participants are only identified by numbers which are destroyed after individual questionnaires have been linked. Spatial data are randomly displaced to ensure individual home locations cannot be located [28].

**Table 1:** Selection of the final study population for each survey included in the analysis.

	Survey								
	Kenya 2009	Lesotho 2014	Malawi 2015	Mozambique 2009	Swaziland 2006	Tanzania 2011	Uganda 2011	Zambia 2013	Zimbabwe 2015
HIV test result available **(N)**	798	791	1657	1836	1195	4234	4453	3487	1978
Data on current education and HIV test result available (Final study population) **(%)**^**1**^	797 (99.9)	791 (100.0)	1657 (100.0)	1679 (91.4)	1185 (99.2)	4155 (98.1)	4453 (100.0)	3438 (98.6)	1978 (100.0)

^1^ The percentages use the number of respondents consented for HIV testing as the denominator.

### Sampling and data collection

The DHS sampling strategy is designed to ensure that when appropriately weighted the study populations are representative at the national level. Data were weighted according to individual sampling probabilities in accordance with the guidance provided by DHS Measure [29]. These sample weights were applied to the data in the derivation of descriptive statistics and all subsequent regression analyses.

### Variables included

The HIV testing protocols varied between sites and are described in each survey report [19–27]. Current school attendance was defined as having attended school at any time during the current school year. Age was stratified into one-year bands. Type of residence was defined as urban or rural for all households in a particular cluster or sample point. Birth history was defined as ever having given birth and ascertained through a self-report from the respondent. The wealth index is a measure of a household's cumulative living standard calculated based on the ownership of selected assets. Households are assigned within each survey into one of five wealth quintiles based on one being the most deprived and five being the least deprived [29]. Current marital status was stratified as never married or ever married. Educational attainment was defined as the highest year of secondary school education achieved. Educational attainment and age were included in the models as continuous independent variables, the other variables were categorical. Age, type of residence, birth history, educational attainment, marital status and household wealth index were considered to be a priori confounders of the association between current school attendance and HIV prevalence.

For Mozambique, data on current schooling was missing for 8.6% (157/1836) of respondents for whom an HIV test result was available; elsewhere the figure was <2% (Table 1).

### Analysis

The individual surveys from the nine DREAMS countries were pooled into one dataset. A logistic regression model was developed to obtain crude and adjusted odds ratios, p-values, and 95% confidence intervals (CI’s) for the association between current school attendance and HIV prevalence. In the adjusted model we controlled for age, type of residence, birth history, educational attainment, marital status and household wealth index. We also included a term representing the interaction between the country and current school attendance and assessed the extent to which the association differed between countries using the Wald test. We pre-determined that if there was significant inter-country variation we would subsequently develop separate logistic regression models for each country, adjusting for the same variables as were included in the model based on pooled data.

To better understand the effect of age on the association between current school attendance and HIV, we plotted the HIV prevalence for those currently in and out of school in each age group. We created additional logistic regression models in which i) an interaction term between age and current school attendance was included and ii) educational attainment was omitted. All analyses were carried out using Stata version 14 [30].

## Results

**Fig 1:**
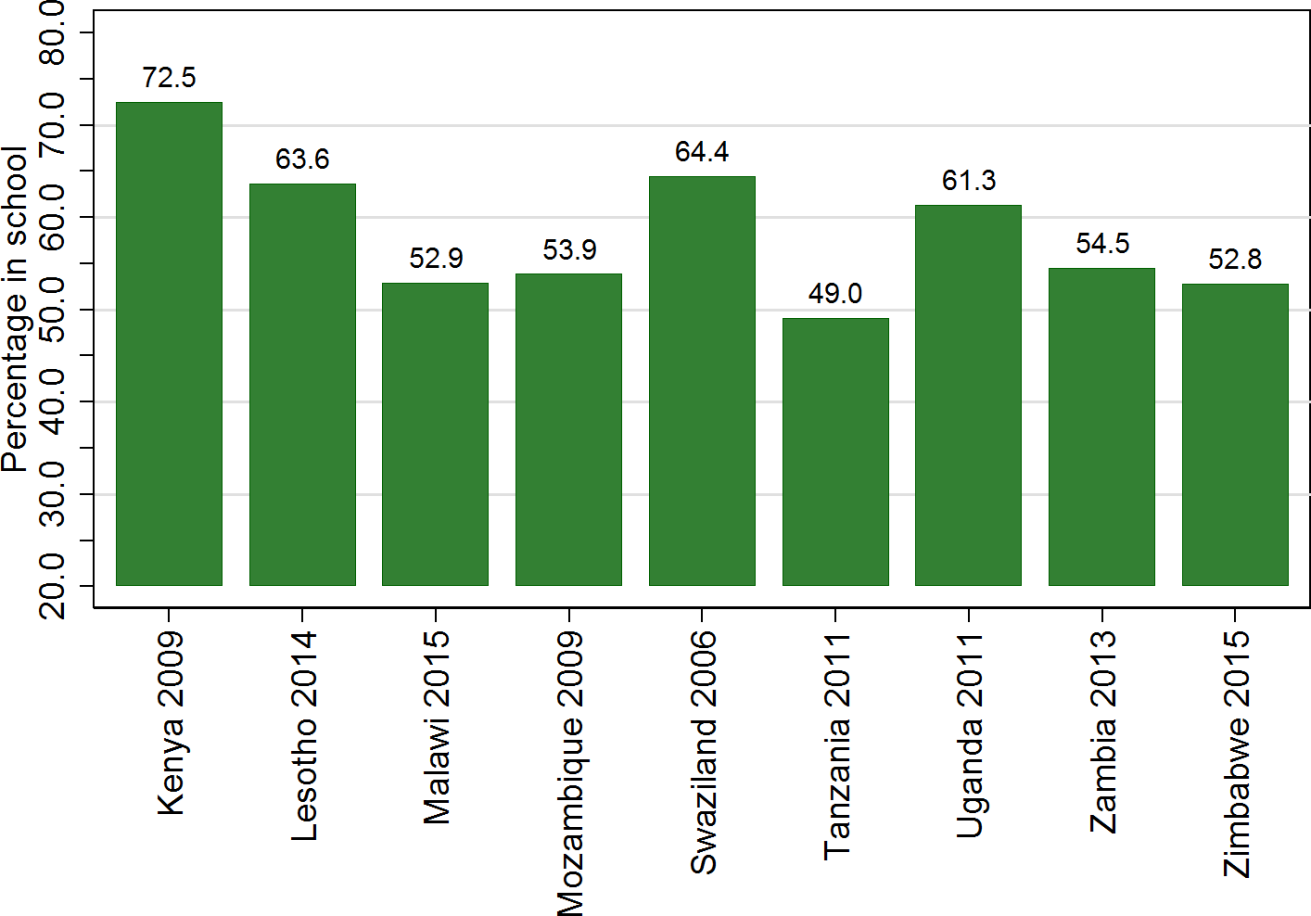
Percentage of women aged 15 to 19 attending school in the current year for each survey included in the analysis. The study population were young women aged 15–19 included in nationally representative Demographic and Health Survey (DHS) Household and AIDS indicator surveys (AIS) carried out in the years indicated in the x-axis labels, for whom HIV test results and data on current education were available. The graph shows the percentage of the study population in each country who had attended school in the year in which the survey was conducted. Data were weighted to account for individual sampling probabilities.

**Fig 2:**
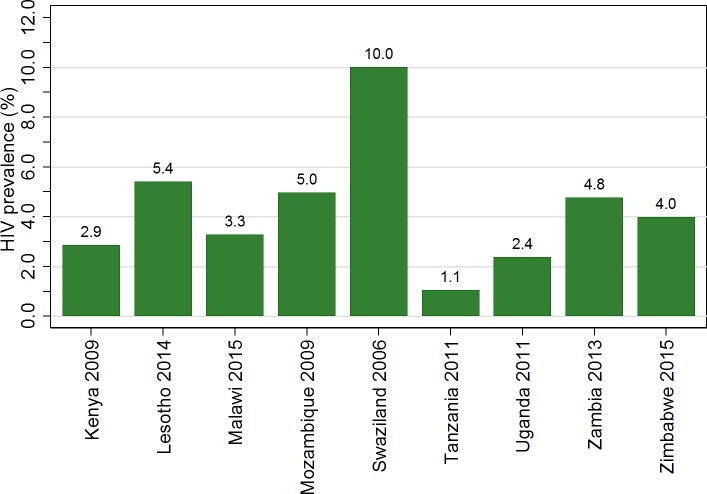
HIV prevalence for women aged 15 to 19 for each survey included in the analysis. The study population were young women aged 15–19 included in nationally representative Demographic and Health Survey (DHS) Household and AIDS indicator surveys (AIS) carried out in the years indicated in the x-axis labels, for whom HIV test results and data on current education were available. The graph shows the percentage of the study population who were found to be HIV positive. Data were weighted to account for individual sampling probabilities. Details of the HIV testing protocol in each site are found in the individual study reports.

The educational and socio-demographic characteristics of young women differed between countries. The percentage in school varied from 50.0% in Tanzania to 72.6% in Kenya (Fig 1). Prevalence of HIV in the study population ranged from 1.0% in Tanzania to 9.8% in Swaziland (Fig 2). The percentage living in an urban area was lowest in Kenya at 14.0% and highest in Zambia at 47.2%. Mean age varied between 16.9 years in Uganda and Zimbabwe and 17.1 years in Kenya and Mozambique. The percentage who had given birth ranged from 10.6% in Tanzania to 23.7% in Zambia. In Zambia, there was little difference in testing by school attendance (93.6% among those in school vs 95.9% among those not in school).

From the logistic regression model developed using pooled data from all surveys there was strong evidence that the association between current school attendance and HIV prevalence differed significantly between countries (p<0.001). Consequently, subsequent analyses were carried out on individual survey datasets, rather than the pooled data.

The crude odds ratios (OR) for individual countries showed a strong association between current school attendance and low HIV prevalence in six of the nine countries (Kenya, Lesotho, Mozambique, Swaziland, Uganda, and Zimbabwe; Table 2), in these countries, the prevalence of HIV among women currently in education was significantly lower than that for those not currently in education. After adjusting for the effect of age, place of residence, educational attainment, wealth index, marital status and birth history, a significant association persisted in Swaziland (aOR: 0.32; 95%CI: 0.17–0.59), Uganda (aOR: 0.48; 95%CI: 0.29–0.80), and Lesotho (aOR: 0.37; 95%CI: 0.17–0.79) (Table 2). In Tanzania there was evidence that the prevalence of HIV was increased for those currently in education (aOR: 3.17; 95%CI 1.15–8.70). The final adjusted models for each country including the adjusted odds ratios and associated confidence intervals are presented in S1 Table.

**Table 2:** HIV prevalence among those in-school and out of school, and crude and adjusted odds ratios for the association between current school attendance and HIV prevalence in individual surveys. Data were weighted to account for individual sampling probabilities. S1 Table presents the adjusted odds ratios and associated confidence intervals for all covariates included in the final adjusted model.

Survey	HIV prevalence for those currently in education % (n/N)	HIV prevalence for those not currently in education % (n/N)	Crude odds ratio (95% Confidence interval)	Adjusted odds ratio ^1^ (95% Confidence interval)	p values for adjusted odds ratios ^1^
**Kenya 2009**	2.0(11/573)	5.2(11/216)	0.37(0.15–0.90)	2.08 (0.66–6.62)	0.213
**Lesotho 2014**	3.7(17/463)	8.3(22/264)	0.42(0.22–0.83)	0.37 (0.17–0.79)	0.011
**Malawi 2015**	3.0(26/881)	3.8(30/790)	0.77(0.36–1.61)	0.77 (0.37–1.63)	0.497
**Mozambique 2009**	3.3(31/927)	6.9(44/640)	0.48 (0.28–0.80)	0.50 (0.22–1.13)	0.094
**Swaziland 2006**	4.6(36/776)	19.6(82/419)	0.20 (0.13–0.30)	0.32 (0.17–0.59)	<0.001
**Tanzania 2011**	1.3(24/2004)	0.7(15/2003)	1.75 (0.81–3.78)	3.17 (1.15–8.70)	0.025
**Uganda 2011**	1.4(39/2729)	3.9(67/1721)	0.36(0.24–0.53)	0.48 (0.29–0.80)	0.005
**Zambia 2013**	4.4(80/1834)	5.4(82/1533)	0.81 (0.55–1.19)	0.92 (0.57–1.48)	0.730
**Zimbabwe 2015**	2.3(24/1057)	5.9(55/946)	0.38 (0.22–0.66)	0.64 (0.31–1.30)	0.214

^1^ Odds ratios adjusted for the effect of for age, type of residence, birth history, marital status, educational attainment and household wealth index

When educational attainment was omitted from the model, there was evidence of a change in the strength of evidence for an association between HIV prevalence and current education in Zimbabwe and Mozambique. The adjusted models for each country including the adjusted odds ratios and associated confidence intervals for the model omitting educational attainment are presented in S2 Table.

**Fig 3:**
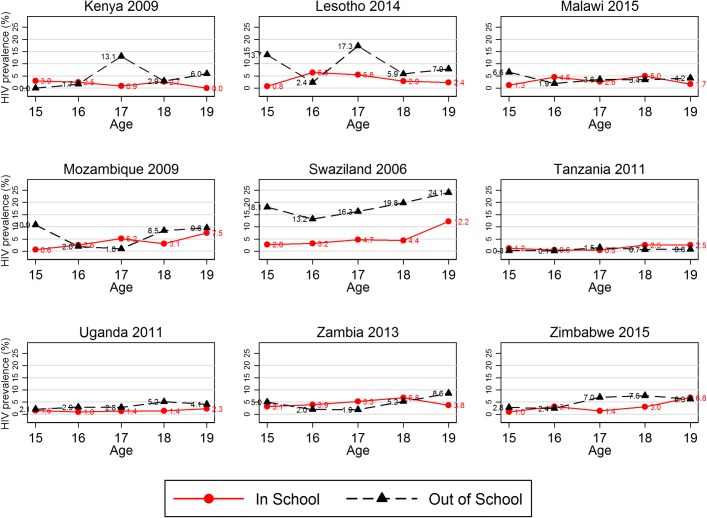
HIV prevalence by current education status for each 1-year age group for each survey included in the analysis. The study population were young women aged 15–19 included in nationally representative Demographic and Health Survey (DHS) Household and AIDS indicator surveys (AIS) carried out in the years indicated in the x-axis labels were available. These graphs show the HIV prevalence in each age group stratified by whether individuals attended school in the year in which the survey occurred. Data were weighted to account for individual sampling probabilities.

Across countries, there was variation in patterns by which education and HIV status varied by age, (Fig 3). There was statistical evidence for an interaction between age and being currently in school in Mozambique (p<0.01), Zambia (p = 0.06), Kenya (p = 0.05) and Lesotho (p = 0.06).

## Discussion

In our analysis of recent nationally representative surveys, conducted between 2006 and 2016, in nine of the ten sub-Saharan African countries targeted by the DREAMS initiative, we found heterogeneity in the association between current school attendance and HIV prevalence among young women. There was evidence for a lower odds of being HIV positive among young women in school in three of the nine countries: Lesotho, Swaziland and Uganda.

Two of the three countries in which being in school was associated with HIV prevalence (Lesotho and Swaziland) also had high HIV prevalence. This suggests that the protective effect of being in school might be greatest where HIV prevalence is highest. There was also some evidence that the association between being in school and HIV prevalence was greatest in countries with high levels of school attendance, as Swaziland, Lesotho and Uganda had the 2nd to 4th highest levels of current school attendance. In Kenya, however, where the level of current school attendance was highest, there was little evidence for an association between school attendance and being HIV positive. There were however only 22 HIV positive individuals in this survey sample, hence there may have been insufficient power to identify an association. A previous paper also using DHS data [9], found that HIV prevalence was higher among more educated young women in Lesotho, Kenya and Zimbabwe whilst in Malawi HIV prevalence was higher amongst those with less education, there was no evidence for this association in Tanzania. The study used educational attainment as its metric rather than currently being in school and included young women aged up to 24 years old in the study population, which may explain the differences with our findings. As there was no consistent trend between countries in the changes of HIV prevalence with age, the variations seen are unlikely to reflect important age-associated differences.

There are some limitations to our analysis. We have shown an association in some countries between being in school and HIV prevalence: we cannot, however, assume that it is causal. There is a possibility that there are other factors that are associated with both the risk of dropping out of school and the risk of having been infected with HIV, this would lead to confounding of the association between these two factors. There is also the possibility of reverse causality with HIV acquisition preceding school drop-out. This could occur, for example, if there were perinatally infected young women in the study population. Inclusion of such individuals who are more likely to be out of school due to illness than their peers, could weaken the evidence for a protective effect of current school attendance against HIV acquisition. The dates of data collection for the surveys included in this study ranged from 2006 to 2016, it is possible that changes in societal norms regarding access to education for females and awareness of HIV prevention among young women occurred over this period. Thus, we should be cautious in making direct comparisons between countries based on these results. When examining the association between HIV and education within countries, by single-year age group, the number of young women within each strata became very low in some cases, which makes our interaction findings difficult to interpret.

It would have been useful to investigate the influence of orphan-hood on the associations and to adjust for its effect in the analyses as previous studies have shown that orphans are a particularly vulnerable group [31]. However, in the surveys analysed, data on the vital status of parents was only collected for those aged 16 and under so such an analysis was not possible for our whole study population. Due to there being missing data in some surveys on consent for HIV testing it was not possible to assess whether the study population of young women aged 15–19 who were tested for HIV differed in key socio-demographic characteristics from the entire population of all young women aged 15–19 selected for HIV testing.

Variations in the association between HIV status and education have also been found in previous studies with a focus on educational attainment rather than current schooling. These studies also found that both the nature and direction of the associations varied between countries and over time [7, 9, 13, 32]. In this study we hypothesised that being in school could be protective as young women spend more time socialising and meeting sexual partners who are likely to be of a similar age and therefore less likely to be HIV positive. In relationships of a similar age the power balance might be more favourable towards the young women, enabling them to negotiate protective sexual practices. A recent study in South Africa indicated that school attendance influenced both the number and age of sexual partners selected by young women [33]. However, HIV prevalence differs significantly across countries and the differences in risk associated with partner age are also likely to differ. The educational context and the proportion of young people in school, along with the difference in school attendance by gender also differs across countries, which means that the social impacts of being in school versus not in school are also likely to vary. Finally, the extent to which young women in or out of school form relationships with older men and the cultural meaning of these relationships will also vary, leading to heterogeneity in the association between current school attendance and HIV prevalence.

The data analysed came from large national surveys using standardised methodologies with limited context specific adaptation. In Zambia there was evidence that the percentage of young women currently attending school differed between those who were tested for HIV and those for whom a test was not carried out. This may have led to a bias in the associations found in that country.

A previous analysis of imputed HIV prevalence values from fourteen nationally representative DHS surveys found the effects of non-response to be small and evidence for bias due to this cause to be weak [34, 35]. Based on this evidence we suggest that survey and testing non-response are unlikely to have influenced the associations seen in this study. We recognise that there are additional sexual and reproductive health related risk factors associated with education that need to be addressed, such as the provision of sanitary products for young women and policies operating in some countries that exclude young women from education if they become pregnant, analysis of these factors was beyond the scope of this study.

On the basis of our study we might expect interventions to keep young women in school to be most effective as a component of the DREAMS package in Swaziland, Lesotho and Uganda and, perhaps, in other countries where the age differences between sexual partners are important risk factors for acquiring HIV among young women.

There are likely to be other contextual factors which differ between countries. For example, if the quality and accessibility of HIV prevention information targeted toward young women varies we would expect this to influence the effect of an increase in socio-cognitive abilities on the risk of acquiring HIV. The protective effect associated with being physically in school would also be influenced by differing culturally associated patterns of sexual behaviour.

There was statistical evidence for an association between being currently in education and an increase in HIV prevalence in Tanzania, however a detailed investigation of the local context would be needed before inferring causality based on this result.

Our findings therefore support the need for the development of targeted interventions based on an understanding of the dynamics of a local HIV epidemic [36, 37]. High quality evaluations of the DREAMS interventions that investigate the evidence for the pathways along which interventions might work and for whom they work, along with the overall impact, will be important for maximising the gains of these initiatives.

## Supporting information

S1 Table Adjusted odds ratios for all covariates for the association between current school attendance and HIV prevalence in individual surveys–Final adjusted model. (PDF)

S2 Table Adjusted odds ratios for all covariates for the association between current school attendance and HIV prevalence in individual surveys–Final adjusted model with educational attainment omitted. (PDF)

## References

[1] UNAIDS. Prevention Gap Report (http://www.unaids.org/en/resources/documents/2016/prevention-gap) 2016.

[2] Patel P, Borkowf CB, Brooks JT, Lasry A, Lansky A, Mermin J. Estimating per-act HIV transmission risk: a systematic review. Aids. 2014;28(10):1509–19. doi: 10.1097/QAD.0000000000000298 24809629 10.1097/QAD.0000000000000298 PMC6195215

[3] Maughan-Brown B, Kenyon C, Lurie MN. Partner Age Differences and Concurrency in South Africa: Implications for HIV-Infection Risk Among Young Women. Aids and Behavior. 2014;18(12):2469–76. doi: 10.1007/s10461-014-0828-6 25047687 10.1007/s10461-014-0828-6 PMC4451824

[4] Ott MQ, Bärnighausen T, Tanser F, Lurie MN, Newell M-L. Age-gaps in sexual partnerships: seeing beyond ‘sugar daddies’. AIDS (London, England). 2011;25(6):861. 10.1097/QAD.0b013e32834344c9 PMC3117250 21358377

[5] Harrison A, Colvin CJ, Kuo C, Swartz A, Lurie M. Sustained high HIV incidence in young women in Southern Africa: social, behavioral, and structural factors and emerging intervention approaches. Current HIV/AIDS Reports. 2015;12(2):207–15. doi: 10.1007/s11904-015-0261-0 25855338 10.1007/s11904-015-0261-0 PMC4430426

[6] DREAMS Core package of Interventions—Summary (https://www.pepfar.gov/documents/organization/269309.pdf) 2016.

[7] Hargreaves JR, Bonell CP, Boler T, Boccia D, Birdthistle I, Fletcher A et al Systematic review exploring time trends in the association between educational attainment and risk of HIV infection in sub-Saharan Africa. Aids. 2008 1 30;22(3):403–14. doi: 10.1097/QAD.0b013e3282f2aac3 18195567 10.1097/QAD.0b013e3282f2aac3

[8] Magadi M, Desta M. A multilevel analysis of the determinants and cross-national variations of HIV seropositivity in sub-Saharan Africa: evidence from the DHS. Health & place. 2011;17(5):1067–83. 21741295 10.1016/j.healthplace.2011.06.004 PMC3248638

[9] Hargreaves JR, Davey C, Fearon E, Hensen B, Krishnaratne S. Trends in Socioeconomic Inequalities in HIV Prevalence among Young People in Seven Countries in Eastern and Southern Africa. PLoS ONE. 2015;10(3):e0121775 doi: 10.1371/journal.pone.0121775 25793608 10.1371/journal.pone.0121775 PMC4368573

[10] De Neve J-W, Fink G, Subramanian S, Moyo S, Bor J. Length of secondary schooling and risk of HIV infection in Botswana: evidence from a natural experiment. The Lancet Global Health. 2015;3(8):e470–e7. doi: 10.1016/S2214-109X(15)00087-X 26134875 10.1016/S2214-109X(15)00087-X PMC4676715

[11] Behrman JA. The effect of increased primary schooling on adult women's HIV status in Malawi and Uganda: Universal Primary Education as a natural experiment. Social science & medicine. 2015;127:108–15. 24985789 10.1016/j.socscimed.2014.06.034

[12] Glynn JR, Carael M, Buve A, Anagonou S, Zekeng L, Kahindo M et al Study Group on Heterogeneity of HIV Epidemics in African Cities. Does increased general schooling protect against HIV infection? A study in four African cities. Tropical medicine & international health. 2004 1;9(1):4–14. 14728602 10.1046/j.1365-3156.2003.01168.x

[13] Hargreaves JR, Morison LA, Kim JC, Bonell CP, Porter JD, Watts C et al The association between school attendance, HIV infection and sexual behaviour among young people in rural South Africa. Journal of Epidemiology & Community Health. 2008 2 1;62(2):113–9. 18192598 10.1136/jech.2006.053827

[14] Baird SJ, Garfein RS, McIntosh CT, Özler B. Effect of a cash transfer programme for schooling on prevalence of HIV and herpes simplex type 2 in Malawi: a cluster randomised trial. The Lancet. 2012;379(9823):1320–9. 10.1016/S0140-6736(11)61709-1 22341825

[15] Pettifor A, MacPhail C, Hughes JP, Selin A, Wang J, Gómez-Olivé FX et al The effect of a conditional cash transfer on HIV incidence in young women in rural South Africa (HPTN 068): a phase 3, randomised controlled trial. The Lancet Global Health. 2016 12 1;4(12):e978–88. doi: 10.1016/S2214-109X(16)30253-4 27815148 10.1016/S2214-109X(16)30253-4 PMC5626439

[16] Stoner MC, Pettifor A, Edwards JK, Aiello AE, Halpern CT, Julien A et al The effect of school attendance and school dropout on incident HIV and HSV-2 among young women in rural South Africa enrolled in HPTN 068. Aids. 2017 9 24;31(15):2127–34. doi: 10.1097/QAD.0000000000001584 28692544 10.1097/QAD.0000000000001584 PMC5599334

[17] Kirby DB, Laris B, Rolleri LA. Sex and HIV education programs: their impact on sexual behaviors of young people throughout the world. Journal of Adolescent Health. 2007;40(3):206–17. doi: 10.1016/j.jadohealth.2006.11.143 17321420 10.1016/j.jadohealth.2006.11.143

[18] Schaefer R, Gregson S, Eaton JW, Mugurungi O, Rhead R, Takaruza A et al Age-disparate relationships and HIV incidence in adolescent girls and young women: evidence from Zimbabwe. AIDS (London, England). 2017 6 19;31(10):1461. 10.1097/QAD.0000000000001506 PMC5457819 28426534

[19] Measure DHS. Demographic and Health Surveys Lesotho 2014, DHS-VI, standard DHS and HIV/Other Biomarkers Datasets. Available from: http://dhsprogram.com/what-we-do/survey/survey-display-462.cfm.

[20] Measure DHS. Demographic and Health Surveys Kenya 2008–2009, DHS-V, standard DHS and HIV/Other Biomarkers Datasets. Available from: http://dhsprogram.com/what-we-do/survey/survey-display-300.cfm.

[21] Measure DHS. Demographic and Health Surveys Malawi 2010, DHS-VI, standard DHS and HIV/Other Biomarkers Datasets. Available from: http://dhsprogram.com/what-we-do/survey/survey-display-333.cfm.

[22] Measure DHS. Demographic and Health Surveys Mozambique 2009, DHS-V, standard DHS and HIV/Other Biomarkers Datasets. Available from: http://dhsprogram.com/what-we-do/survey/survey-display-322.cfm.

[23] Measure DHS. Demographic and Health Surveys Swaziland 2006–07, DHS-V, standard DHS and HIV/Other Biomarkers Datasets. Available from: http://dhsprogram.com/what-we-do/survey/survey-display-259.cfm.

[24] Measure DHS. Demographic and Health Surveys Tanzania 2011–12, DHS-VI, standard DHS and HIV/Other Biomarkers Datasets. Available from: http://dhsprogram.com/what-we-do/survey/survey-display-393.cfm.

[25] Measure DHS. Demographic and Health Surveys Uganda 2011, DHS-VI, standard DHS and HIV/Other Biomarkers Datasets. Available from: http://dhsprogram.com/what-we-do/survey/survey-display-373.cfm.

[26] Measure DHS. Demographic and Health Surveys Zambia 2013–14, DHS-VI, standard DHS and HIV/Other Biomarkers Datasets. Available from: http://dhsprogram.com/what-we-do/survey/survey-display-406.cfm.

[27] Measure DHS. Demographic and Health Surveys Zimbabwe 2015, DHS-VII, standard DHS and HIV/Other Biomarkers Datasets. Available from: http://dhsprogram.com/what-we-do/survey/survey-display-475.cfm.

[28] USAID DHS Program—Protecting the Privacy of DHS Survey respondents - https://dhsprogram.com/What-We-Do/Protecting-the-Privacy-of-DHS-Survey-Respondents.cfm.

[29] Guide to DHS statistics - https://dhsprogram.com/pubs/pdf/DHSG1/Guide_to_DHS_Statistics_29Oct2012_DHSG1.pdf.

[30] StataCorp. 2015 Stata Statistical Software: Release 14. College Station, TX: StataCorp LP.

[31] Operario D, Underhill K, Chuong C, Cluver L. HIV infection and sexual risk behaviour among youth who have experienced orphanhood: systematic review and meta-analysis. Journal of the International AIDS Society. 2011;14(1):25. 21592368 10.1186/1758-2652-14-25 PMC3114697

[32] Hargreaves JR, Davey C, White RG. Does the ‘inverse equity hypothesis’ explain how both poverty and wealth can be associated with HIV prevalence in sub-Saharan Africa? Journal of Epidemiology and Community Health. 2013;67(6):526–9. doi: 10.1136/jech-2012-201876 23235546 10.1136/jech-2012-201876

[33] Stoner MC, Edwards JK, Miller WC, Aiello AE, Halpern CT, Julien A et al Effect of Schooling on Age-Disparate Relationships and Number of Sexual Partners Among Young Women in Rural South Africa Enrolled in HPTN 068. JAIDS Journal of Acquired Immune Deficiency Syndromes. 2017 12 15;76(5):e107–14. doi: 10.1097/QAI.0000000000001544 28902703 10.1097/QAI.0000000000001544 PMC5680112

[34] Mishra V, Vaessen M, Boerma J, Arnold F, Way A, Barrere B et al HIV testing in national population-based surveys: experience from the Demographic and Health Surveys. Bulletin of the World Health Organization. 2006 7 10;84:537–45. 16878227 10.2471/blt.05.029520 PMC2627390

[35] Mishra V, Barrere B, Hong R, Khan S. Evaluation of bias in HIV seroprevalence estimates from national household surveys. Sexually Transmitted Infections. 2008;84(Suppl 1):i63–i70. 18647869 10.1136/sti.2008.030411 PMC2569831

[36] Wilson D, Halperin DT. “Know your epidemic, know your response”: a useful approach, if we get it right. The Lancet. 2008;372(9637):423–6. 10.1016/S0140-6736(08)60883-1 18687462

[37] World Health Organization—Guidelines for second generation HIV surveillance: An update: Know your epidemic. Geneva: 2013 9241505826. 24049865

