# Supplementary material for: The association between being currently in school and HIV prevalence among young women in nine eastern and southern African countries
Source: PLoS One. 2018 Jun 20;13(6):e0198898. doi: 10.1371/journal.pone.0198898 (PMC6010266; doi:10.1371/journal.pone.0198898)
Supplement: S1 Table — (PDF) [file pone.0198898.s001.pdf]

		Adjusted Odds ratios with 95% Confidence intervals								
**Variable**	**Category (if applicable)**	**Kenya 2009**	**Lesotho 2014**	**Malawi 2015**	**Mozambique 2009**	**Swaziland 2006**	**Tanzania 2011**	**Uganda 2011**	**Zambia 2013**	**Zimbabwe 2015**
**Current School attendance**	**No**	1	1	1	1	1	1	1	1	1
	**Yes**	**2.08 (0.66-6.62)**	**0.37 (0.17-0.79)**	**0.77 (0.37-1.63)**	**0.50 (0.22-1.13)**	**0.32 (0.17-0.59)**	**3.17 (1.15-8.70)**	**0.48 (0.29-0.80)**	**0.92 (0.57-1.48)**	**0.64 (0.31-1.30)**
**Age (Years from 15-19)**		0.87 (0.54-1.41)	1.02 (0.74-1.39)	1.02 (0.82-1.27)	1.35 (0.99-1.85)	1.33 (1.10-1.61)	1.28 (0.89-1.84)	1.04 (0.85-1.27)	1.23 (1.04-1.47)	1.57 (1.22-2.02)
**Place of residence**	**urban**	1	1	1	1	1	1	1	1	1
	**rural**	1.23 (0.34-4.50)	0.59 (0.25-1.43)	0.57 (0.24-1.34)	0.80 (0.36-1.79)	0.88 (0.48-1.61)	0.72 (0.22-2.38)	1.56 (0.62-3.95)	0.54 (0.32-0.92)	1.13 (0.34-3.68)
**Socioeconomic position**	**poorest**	1	1	1	1	1	1	1	1	1
	**poorer**	0.52 (0.11-2.60)	0.56 (0.14-2.29)	0.71 (0.28-1.84)	0.61 (0.09-4.20)	1.04 (0.48-2.26)	0.76 (0.20-2.83)	0.69 (0.31-1.53)	1.06 (0.48-2.37)	0.88 (0.35-2.18)
	**middle**	2.75 (0.71-10.68)	0.80 (0.23-2.79)	0.74 (0.31-1.76)	0.73 (0.16-3.35)	0.88 (0.41-1.91)	1.14 (0.34-3.79)	1.22 (0.63-2.37)	1.62 (0.75-3.52)	1.47 (0.65-3.33)
	**richer**	3.54 (1.17-10.67)	1.02 (0.32-3.31)	0.99 (0.41-2.41)	2.67 (0.61-11.73)	1.33 (0.65-2.73)	0.99 (0.27-3.56)	1.06 (0.52-2.14)	1.34 (0.58-3.12)	0.97 (0.33-2.90)
	**richest**	0.11 (0.02-0.86)	0.91 (0.24-3.44)	1.09 (0.44-2.74)	3.68 (0.73-18.42)	1.33 (0.56-3.17)	3.03 (0.72-12.71)	1.75 (0.68-4.51)	1.37 (0.53-3.55)	1.21 (0.28-5.35)
**Birth history**	**Never given birth**	1	1	1	1	1	1	1	1	1
	**At least one birth**	2.38 (1.12-5.07)	0.82 (0.29-2.30)	0.88 (0.42-1.84)	0.54 (0.19-1.56)	0.78 (0.41-1.47)	1.02 (0.31-3.34)	1.76 (0.87-3.52)	0.99 (0.57-1.71)	0.99 (0.48-2.07)
**Current Marital status**	**Never married**	1	1	1	1	1	1	1	1	1
	**Ever married**	14.98 (6.00-37.42)	0.89 (0.33-2.36)	0.79 (0.37-1.67)	2.38 (0.78-7.25)	2.37 (1.24-4.54)	3.71 (1.44-9.55)	1.34 (0.67-2.68)	1.05 (0.57-1.93)	0.67 (0.29-1.55)
**Educational attainment (Years of secondary education completed)**		0.66 (0.40-1.09)	0.99 (0.76-1.29)	0.86 (0.63-1.18)	0.96 (0.85-1.08)	0.87 (0.73-1.03)	0.88 (0.67-1.14)	1.06 (0.92-1.23)	1.02 (0.89-1.16)	0.73 (0.60-0.90)

Supplemental table S1 - Adjusted odds ratios for all covariates for the association between current school attendance and HIV prevalence in individual surveys. Data were weighted to account for individual sampling probabilities. Odds ratios adjusted for the effect of for age, type of residence, birth history, marital status, educational attainment and household wealth index.
